# Synchrony Between Default-Mode and Sensorimotor Networks Facilitates Motor Function in Stroke Rehabilitation: A Pilot fMRI Study

**DOI:** 10.3389/fnins.2020.00548

**Published:** 2020-06-16

**Authors:** Changwei W. Wu, Shang-Hua N. Lin, Li-Ming Hsu, Shih-Ching Yeh, Shiao-Fei Guu, Si-Huei Lee, Chun-Chuan Chen

**Affiliations:** ^1^Graduate Institute of Mind, Brain and Consciousness, College of Humanities and Social Sciences, Taipei Medical University, Taipei, Taiwan; ^2^Brain and Consciousness Research Center, College of Humanities and Social Sciences, Shuang-Ho Hospital, Taipei Medical University, Taipei, Taiwan; ^3^Institute of Neuroscience, National Yang-Ming University, Taipei, Taiwan; ^4^Biomedical Research Imaging Center, School of Medicine, University of North Carolina at Chapel Hill, Chapel Hill, NC, United States; ^5^Department of Computer Science and Information Engineering, National Central University, Taoyuan, Taiwan; ^6^Department of Physical Medicine and Rehabilitation, Taipei Veterans General Hospital and National Yang-Ming University, Taipei, Taiwan; ^7^Department of Biomedical Sciences and Engineering, National Central University, Taoyuan, Taiwan

**Keywords:** stroke, rehabilitation, neuroplasticity, motor, resting-state functional MRI, functional connectivity

## Abstract

Stroke is the most common cause of complex disability in Taiwan. After stroke onset, persistent physical practice or exercise in the rehabilitation procedure reorganizes neural assembly for reducing motor deficits, known as neuroplasticity. Neuroimaging literature showed rehabilitative effects specific to the brain networks of the sensorimotor network (SMN) and default-mode network (DMN). However, whether between-network interactions facilitate the neuroplasticity after stroke rehabilitation remains a mystery. Therefore, we conducted the longitudinal assessment protocol of stroke rehabilitation, including three types of clinical evaluations and two types of functional magnetic resonance imaging (fMRI) techniques (resting state and grasp task). Twelve chronic stroke patients completed the rehabilitation protocol for at least 24 h and finished the three-time assessments: before, after rehabilitation, and 1 month after the cessation of rehabilitation. For comparison, age-matched normal controls (NC) underwent the same fMRI evaluation once without repeated measure. Increasing scores of the Fugl–Meyer assessment (FMA) and upper extremity performance test reflected the enhanced motor performances after the stroke rehabilitation process. Analysis of covariance (ANCOVA) results showed that the connections between posterior cingulate cortex (PCC) and iM1 were persistently enhanced in contrast to the pre-rehabilitation condition. The interactions between PCC and SMN were positively associated with motor performances. The enhanced cross-network connectivity facilitates the motor recovery after stroke rehabilitation, but the cross-network interaction was low before the rehabilitation process, similar to the level of NCs. Our findings suggested that cross-network connectivity plays a facilitatory role following the stroke rehabilitation, which can serve as a neurorehabilitative biomarker for future intervention evaluations.

## Introduction

Stroke is the most common cause of complex disability in Taiwan (Hsieh and Chiou, 2014). Most stroke patients survive the initial insult but are left with cognitive impairments, such as movement, sensation, language, memory, and emotion (Carey, 2012). Therefore, rehabilitation protocols play essential roles in post-stroke intervention to lessen the disabilities and regain their quality of life. However, rehabilitative neuroplasticity is a time-dependent process, and the efficacy is unique to each patient (Kolb et al., 2010). Therefore, successful rehabilitative training and an effective evaluation approach are of great importance for post-stroke healthcare, assisting patients returning to meaningful daily activities. Currently, clinical guidelines for stroke rehabilitation are available (Carey, 2012), but the typical evaluation of stroke rehabilitation relies on the patients’ and physicians’ subjective opinions on patients’ motor improvements. Subjective evaluations on behavioral performances may not reflect the ongoing neurophysiological progress following neurorehabilitation. Henceforth, current neuroimaging methodologies, such as functional magnetic resonance imaging (fMRI), offer the possibility to frame post-stroke neuroplasticity objectively (Carey and Seitz, 2007; Liu et al., 2017). These approaches are especially useful when the neural mechanisms of post-stroke recovery over time reflect different pathophysiological phases after ischemic stroke (Carey and Seitz, 2007).

Pathophysiological abnormalities in brain functions can be evaluated by fMRI through two different strategies: brain activity on task engagement and brain connectivity in a resting state. Compared with the limb movement performance in healthy participants, higher contra-lesional motor activity was observed in stroke patients (Enzinger et al., 2008; Carey L. et al., 2011), suggesting the reduced inhibition to the contra-lesional motor cortex during task engagements (Nowak et al., 2009). The task-evoked brain activity was proved to be associated with behavioral performances (Carey L. M. et al., 2011). In contrast, the resting state functional connectivity (RSFC) provides another viewpoint of the brain integrity during a spontaneous state without task engagements (Biswal et al., 1995). The inter-hemispheric RSFC may act as the cognitive reserve in support of task events, and the ipsilesional RSFC usually associates with unilateral neuropathologies in recent studies (Fan et al., 2015). Literature also showed that inter-hemispheric RSFC of sensorimotor network (SMN) was disrupted after stroke onset (Carter et al., 2010; van Meer et al., 2010; Zhang et al., 2016), associated with the reduction of limb movements and gait (Enzinger et al., 2008; van Meer et al., 2012).

Beyond the SMN, the post-stroke connectivity loss in the default-mode network (DMN) and frontoparietal network (FPN) were also found to be associated with cognitive impairments in literature (Tuladhar et al., 2013; Li et al., 2014; Liu et al., 2017). A previous study showed that decreased RSFC of DMN was associated with cognitive decline in stroke patients (Liu et al., 2014), and the attention deficits following stroke onset were associated with FPN (Lincoln et al., 2000; Bajaj et al., 2015). Furthermore, longitudinal RSFC studies disclosed plausible neural reorganizations after stroke onset. For example, Miao et al. observed the progressive inter-hemispheric RSFC normalization in the SMN (van Meer et al., 2012), and the DMN connectivity was restored 3 months after stroke (Park et al., 2014). However, although the brain functionality is accomplished by internetwork integrity as a whole unit, a majority of literature focused on connectivity disruptions of one specific network after stroke (Zhang et al., 2016; Veldsman et al., 2018). Most recent studies demonstrated that inter-network connections serve important roles in cognitive functions. For example, Wang et al. (2014) stated that subcortical stroke affects not only the intra-network connectivity but also the internetwork RSFC. Lam et al. (2018) described the coupling between contra-lesional motor and FPNs correlated with the post-stroke motor outcome. To date, these studies reported the internetwork interactions between stroke patients and controls, yet evaluate the cross-network interactions along the neurorehabilitation process. Therefore, it remains elusive whether the cross-network interactions interfere or facilitate brain reorganizations along the stroke rehabilitation process.

Targeting this issue, we hypothesized that the internetwork connectivity between SMN, DMN, and FPN contributes to regaining of motor functions along the stroke rehabilitation process. To attain this goal, we recruited 15 subcortical stroke patients for longitudinal fMRI assessments. We performed longitudinal assessments three times along the rehabilitative intervention (pre-rehab, post-rehab, 1 month follow up). For each assessment, the patients performed a grasp task and resting state fMRI to evaluate both brain activity and functional connectivity.

## Materials and Methods

### Clinical Assessments

A total of 15 chronic stroke patients were enrolled in this study, and 12 normal controls (NCs) were also included as an age-matched control group. All participants provided written consent forms under the supervision of the Institutional Review Board of Taipei Veterans General Hospital (IRB number: 2013-05-018A). Patients were clinically assessed from the chronic stroke and rehabilitation services at the Taipei Veterans General Hospital, Physical Medicine and Rehabilitation Department. Patients were recruited if they meet the inclusion criteria: (1) clinical diagnosis of unilateral stroke infarct confirmed by a physician based on neurological examinations and brain imaging (MRI or CT scan); (2) aged between 25 and 85 years; (3) Brunnstrom stage II–V over the proximal and distal part of the upper extremity on the affected side; (4) no cognitive dysfunction, measured by the Mini-Mental State Exam (≧24; suggested by Crum et al., 1993); (5) in chronic phase after prior stroke onset (≧6 months); and (6) willing/able to participate and having signed an informed consent form. Exclusion criteria were as follows: (1) unstable vital sign; (2) irreversible contracture over any of the joints of the affected upper extremity; (3) history of surgery, fracture, arthritis, pain, or any other complications that might influence the recovery of upper extremity function, such as aphasia, apraxia, and neglect; (4) having spasticity as measured using the modified Ashworth scale (score > 2); (5) having post-stroke seizure; (6) heart attack within 3 months post-stroke; (7) cortical lesions in any of the five core motor areas of interest, including the bilateral primary motor cortex (M1), the bilateral premotor area, and the supplementary motor area (SMA); and (8) with metal implants. Uniquely, six participants reported the use of medications during the rehabilitation period: medication for hypertension (*n* = 5, Valsartan and Olmesartan), medication for antiplatelet (*n* = 1, Cloropidol), and medication for seizure prevention (*n* = 4, Baclofen).

### Stroke Rehabilitation and Behavioral Assessments

After receiving the clinical assessment, all stroke patients received rehabilitation treatments with a total duration of 24 h. Patients received 1 h of rehabilitation training at an intensity of three times per week over 8 weeks (total 24 sessions). The treatment program contains the motor-skill exercises that can improve patients’ muscle strength and coordination and the range-of-motion therapy that can ease the patient’s spasticity and help the patient regain range of motion. The rehabilitative plan was individualized according to the situation of each participant by Taipei Veterans General Hospital. For all patients, the time interval between two consecutive training sessions was longer than 24 h to avoid fatigue. The rehabilitative treatments were adjusted according to the situation of the participants by Taipei Veterans General Hospital.

For evaluating the degree of motor impairments, we recorded three behavior scores before the treatment (Pre), after the treatment (Post), and at 1-month follow-up (Follow) after treatment. Fugl–Meyer assessment (FMA) of physical performance is a tool for quantitative assessment design based on the recovery process of the stroke patients (Fugl-Meyer et al., 1975), assessing the motor function, motor coordination, speed, balance function, sensation, etc. The therapist recorded the corresponding score based on the performance of stroke patients between 0 and 66, where high scores correspond to good motor performance for all three assessments. FMA was selected to evaluate the upper limb motor function because it is highly recommended for measuring the outcomes of stroke patients (Bushnell et al., 2015). The wolf motor function test (WMFT) is an assessment of upper limb function for patients based on the performing integrity and fluency of the specified action (Wolf et al., 2001). WMFT contains 15 tasks related to daily-life actions, such as turning keys, taking a basket, flipping cards, etc. Each task scores between 0 and 5 (up to 75 points). The upper extremity performance test for the elderly (TEMPA) is an assessment for daily-life motor tasks with four divisions, ranging from -138 to 0 points (Desrosiers et al., 1995). The closer to 0, the better the performance. The therapist recorded the corresponding score based on the motor performance of stroke patients, and high scores correspond to good motor performance for all three assessments.

### Experimental Design and fMRI Protocol

The fMRI protocol consisted of both resting state and movement-related tasks, which were conducted three times in a repeated-measure design (Pre, Post, and Follow) for the stroke patients and performed once on NCs. Due to the limited space inside the MRI scanner, we chose grasp as the target motion task in preventing unnecessary motion during MRI acquisition. All participants practiced the grasp task once outside the MRI scanning room to ensure their compliance. The visual instructions and stimuli were given using the projector with mirror settings. In the session of resting state, we instructed the participants to keep their eyes open, keep head position still, not fall asleep, and not think of anything in particular, resting for 6 min. At the end of the resting session, the participants were asked to press the alarm ball in prevention of drifting into sleep. In the session of the grasp task, participants were instructed to perform a block-design task: eight cycles of 20-s grasp–release actions followed by 20 s of resting condition with an initial wait of 10 s rest, lasting for 6 min (180 measurements) in total. The participants were instructed to grasp with the left hand for the first two cycles and then right-hand grasp, alternately switching hands every two cycles (Supplementary Figure S1).

The fMRI data were acquired using a Siemens TIM Trio 3T MRI scanner with a 12-channel head coil. For fMRI scans, we applied a gradient-echo echo planar imaging sequence (TR = 2000 ms; TE = 35 ms; flip angle = 90°, 31 slices, matrix size = 64 × 64; FOV = 220 mm; voxel size = 3.4 × 3.4 × 4 mm^3^). The scanning slab was aligned along the anterior commissure and posterior commissure line. A high-resolution T1-weighted anatomical image was also acquired by an MP-RAGE sequence for registration purposes (TR = 1900 ms; TE = 2.26 ms; flip angle = 9°; 176 slices; FOV = 224 × 256 mm). The field-map images were also acquired for EPI geometric corrections. The T_2_-FLAIR images were acquired for localization of the lesion site and for special normalization on stroke-patient datasets using the automated lesion identification (ALI) toolbox (Seghier et al., 2008). The datasets for this study are available on request to the corresponding author.

### fMRI Analysis

Preprocessing of fMRI data was performed using statistical parametric mapping (SPM12) and analysis of functional neuro-images (AFNI) (Cox, 1996). We checked the lesion locations in the beginning and flipped left to right for patients who had a lesion site at the left hemisphere (regarding the right-hand side as the affected side in the following analysis). Then the field-map correction was applied to unwarp distorted datasets. The motion correction and the co-registration between T1-weighted images and functional datasets were performed. The two-step segmentation normalization was used to segregate six tissue types: gray matter, white matter, cerebrospinal fluid (CSF), skull, soft tissue, outside head, and then transformed into the Montreal Neurological Institute (MNI) space via DARTEL normalization (Seghier et al., 2008; Ripollés et al., 2012). Last, we smoothed all images with a full-width half-maximum of 8 mm. After the preprocessing, the grasp-task data underwent the regression analysis using the general linear model incorporating nuisance regressors (baseline, linear trend, head motion). The grasp-task paradigm was convolved with a canonical hemodynamic response function and used as the main effect for both affected and unaffected hands/hemispheres. The group-level activation maps were generated based on beta maps for both stroke patients and NC.

An additional three steps for the RS-fMRI data included (1) the CSF and white matter signals extracted from the fMRI datasets were taken as the nuisance regressor, (2) linear detrend, and (3) band-pass filtering between 0.01 and 0.08 Hz. To evaluate the functional connectivity of the three networks (DMN, FPN, and SMN), we adopted a seed-based correlation strategy. Spherical seeds (4 mm in radius) were applied for each of four networks: (1) posterior cingulate cortex (PCC) [0 -53 26] for DMN (Van Dijk et al., 2010), (2) ipsilesional middle frontal gyrus [45 29 32] for affected FPN (Lin et al., 2014), (3) contra-lesional primary motor cortex (cM1) [36 −25 57] for unaffected SMN (Van Dijk et al., 2010), and (4) ipsilesional primary motor cortex (iM1) [−36 −25 57] for affected SMN. All the coordinates were based on the MNI system, and the affected side for the NC group was set as the right-hand side. Connectivity strengths were evaluated through the temporal correlation between the seed signal and the signal from every other voxel. Subsequently, we transformed the correlation coefficients into a *z* value by Fisher-*Z* transformation for group analysis.

### Statistics

For stroke patients, we performed a one-way repeated-measure analysis of covariance (ANCOVA) to control age, gender, education, and time since the stroke onset. The ANCOVA was applied to FMA/WMFT/TEMPA scores, task-fMRI (affected-hand grasp and unaffected-hand grasp), and RS-fMRI indices (DMN, FPN, and SMN) for evaluating the rehabilitative effect, and corresponding *post hoc* tests were used to reveal the significant difference between time points. In the imaging statistics, we presented the one-sample *T*-maps and ANCOVA *F*-maps with a 3dClustSim corrected *p* < 0.05 with autocorrelation function (ACF). Subsequently, we applied regions of interest (ROIs) of the RSFC and grasp activations from the NC group (uncorrected *p* < 10^–5^) to the longitudinal datasets along stroke rehabilitation and examine the associations between motor-function assessments and imaging indices.

## Results

### Behavior Improvements After Stroke Rehabilitation

**FIGURE 1:**
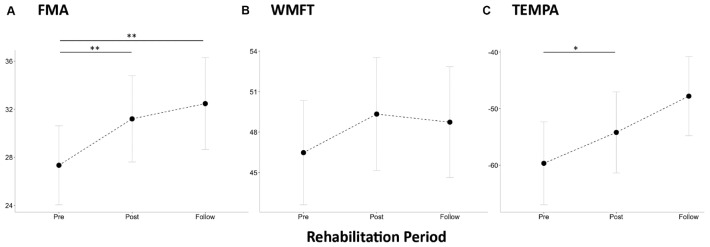
Average behavioral assessments and brain activity/connectivity maps in stroke patients (*n* = 12 with longitudinal assessments) and normal control (*n* = 12). **(A)** Fugl–Meyer assessment of physical performance (FMA), **(B)** Wolf motor function test (WMFT), and **(C)** upper extremity performance test for the elderly (TEMPA). Single asterisk sign denotes statistical significance *p* < 0.05, and double asterisk sign denotes *p* < 0.01 with Bonferroni correction.

Among the collected patients, three stroke patients were excluded for their post-stroke subacute phase, and 12 patients with chronic stroke completed the three longitudinal fMRI assessments. The demographic information is listed in Supplementary Table S1, where the time duration after stroke onset ranged from 6 to 24.7 months among patients. Considering their motor performances, the repeated-measure ANCOVA showed statistical significance of FMA (Figure 1A) and TEMPA (Figure 1C) scores along the rehabilitation process (FMA_Pre__:__Post__:__Follow_ = 26.8:29.3:30.3, *F*_2,22_ = 6.3, *p* < 0.007; TEMPA_Pre:Post:Follow_ = −59.8:−55.3:−50.2, Huynh- Feldt-corrected *F*_1.4,15.5_ = 7.9, *p* < 0.008), but WMFT (Figure 1B) did not show a significant difference (WMFT_Pre:Post:Follow_ = 45.5:48.0:46.9, *F*_2,22_ = 6.3, *p* < 0.007; TEMPA_Pre:Post:Follow_ = 45.5:48.0:46.9, *F*_2,22_ = 1.30, *p* = 0.294). The *post hoc* comparison demonstrated significant FMA enhancements after the rehabilitation process (Pre vs. Post and Follow, Bonferroni-corrected *p* < 0.05), and TEMPA was enhanced between Pre and Post states (Bonferroni-corrected *p* = 0.03). The detailed information is listed in Supplementary Table S2.

### Brain Activity of Grasp Task vs. Within-Network Integrity of Resting State

**FIGURE 2:**
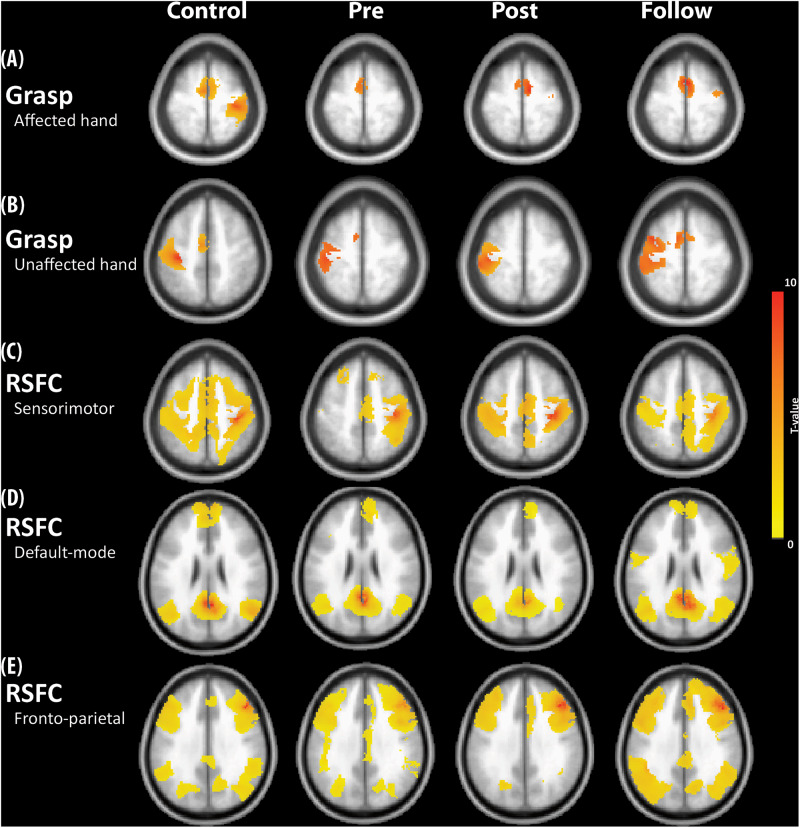
**(A)** Activity of grasp task using the affected hand. **(B)** Activity of grasp task using the unaffected hand. **(C)** RSFC of sensorimotor network (SMN). **(D)** RSFC of default-mode network (DMN). **(E)** RSFC of frontoparietal network (FPN). The seeds were placed at the ipsilesional side of the brain. Error bar denotes the standard error.

**FIGURE 3:**
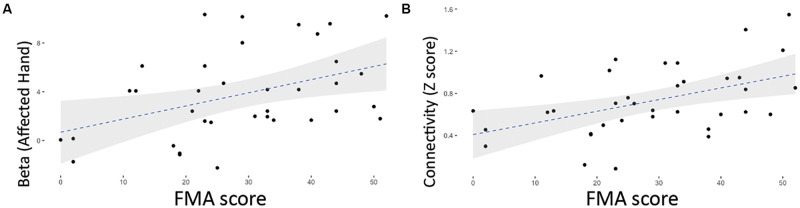
Positive relationship between FMA scores and the brain functional indices in the 12 stroke patients. **(A)** Association between FMA and the grasp-task activity (by affected hand) (*r* = 0.423, *p* = 0.01). **(B)** Positive relationship between FMA and the functional connectivity of contralesional M1 (seeding at ipsilesional M1) (*r* = 0.478, *p* = 0.003).

Figures 2A,B show the one-sample *t* maps of the grasp task in the affected and unaffected sides for all participants, respectively, and the statistical criteria were set as 3dClustSim-corrected *p* < 0.05 (uncorrected *p* < 10^–4^ with ACF and cluster threshold of 20 voxels). In the unaffected side, the grasp-induced brain activities localized at contra-lesional M1 and SMA regions, resembling that of NC. However, in the affected side, the grasp induced brain activities over only the SMA region in the Pre state and over the contra-lesional M1 and SMA in both Post and Follow. Figures 2C–E show the group-level functional connectivity of SMN, DMN, and FPN, respectively, all seeding at the ipsilesional side (right-hand side in NC) under the same statistical threshold. Compared with the connectivity patterns in the NC, DMN and FPN seemed unchanged across three repeated measures. DMN and FPN in stroke patients resembled that in NC to a great degree across the three sections only the SMN in stroke patients was mainly unilateral in the Pre-state, but became bilateral in Post and Follow conditions. Furthermore, both the magnitudes of brain activity (beta value) and the brain connectivity (*z* score) moderately reflected the motor performances of FMA scores along the rehabilitation process. The recovered beta values of the grasp task (by affected hand) in Figure 3A show a positive relationship with the FMA scores (*r*^2^ = 0.18, *p* < 0.01), where the bilateral SMN connectivity strength (seeding at ipsilesional M1) in Figure 3B also demonstrates significant association with the FMA scores (*r*^2^ = 0.23, *p* < 0.01).

### Internetwork Connectivity Between SMN and DMN in Stroke Rehabilitation

**FIGURE 4:**
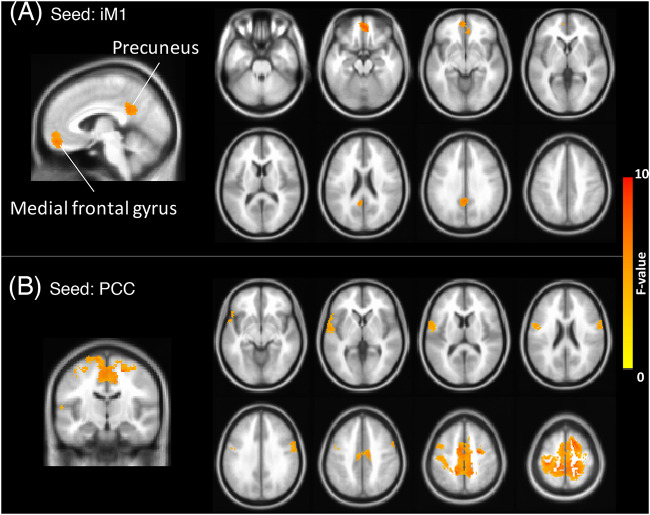
Group-based ANCOVA results across three longitudinal assessments in stroke patients (3dClustSim corrected *p* < 0.05). **(A)** RSFC of SMN seeding at iM1, highlighting medial frontal gyrus and precuneus. **(B)** RSFC of DMN seeding at PCC, highlighting primary motor cortices, and supplementary motor area. Factors of age, gender, education, and time after stroke onset were controlled as covariates.

**FIGURE 5:**
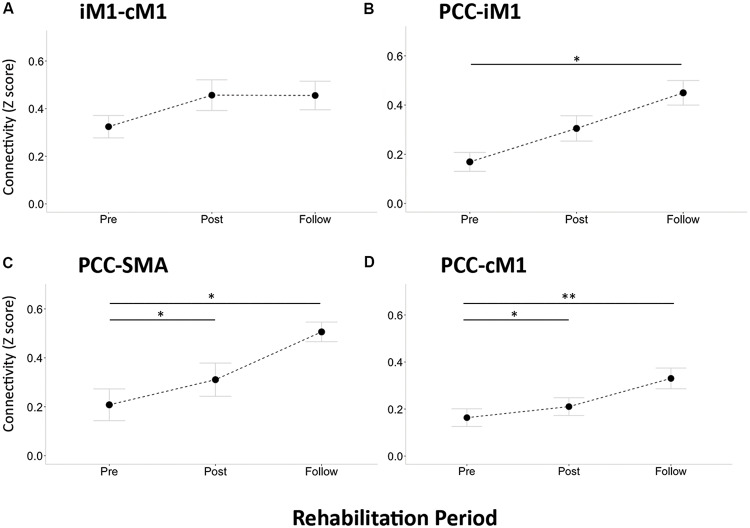
Regions of interest (ROIs)-based RSFC across longitudinal assessments in stroke patients. **(A)** Connection between iM1 and cM1. **(B)** Connection between PCC and iM1. **(C)** Connection between PCC and SMA. **(D)** Connection between PCC and cM1. Connectivity strength was quantified by *z* score. Error bar denotes the standard error. Single asterisk sign denotes statistical significance *p* < 0.05, and double asterisk sign denotes *p* < 0.01 with FDR correction.

To estimate the internetwork interactions along the rehabilitation process, Figure 4 displays the ANCOVA results of stroke patients across the three time points (3dClustSim-corrected *p* < 0.05, uncorrected *p* < 0.01 with ACF and cluster threshold of 300 voxels). SMN connectivity patterns (seeding at iM1) had rehabilitation effects on precuneus and medial frontal gyrus along the rehabilitation process (Figure 4A), and DMN (seeding at PCC) interacted with the bilateral M1, SMA, and premotor cortex (Figure 4B). However, the FPN did not show significant alterations along the rehabilitation process in ANCOVA; henceforth, we simply focus on the SMN–DMN interactions in the following analysis. Subsequently, we performed the ROI analysis on the internetwork interactions within DMN/SMN regions prescribed from the NC group (*post hoc* tests adjusted with multiple comparison). Figure 5A shows the insignificant changes of intra-network connectivity strengths (*z* scores) between bilateral M1s across three time points (*F*_2,22_ = 1.55, *p* > 0.2), and Figures 5B–D show the intra-network connectivity strengths between PCC and SMN-related regions, demonstrating that PCC had enhanced connections to SMN-related regions (*F*_2,22_ > 5.1, *p* < 0.02) in the Post and Follow states. In contrast, the NC group did not present observable PCC-SMN connections, and the enhanced PCC-SMN connectivity after stroke rehabilitation (Post and Follow) was significantly higher than the connectivity level of the NC group (two-sample *t*-test *p* < 0.05, data not shown).

### Associations Between DMN–SMN Connectivity and Behavioral Performance

**FIGURE 6:**
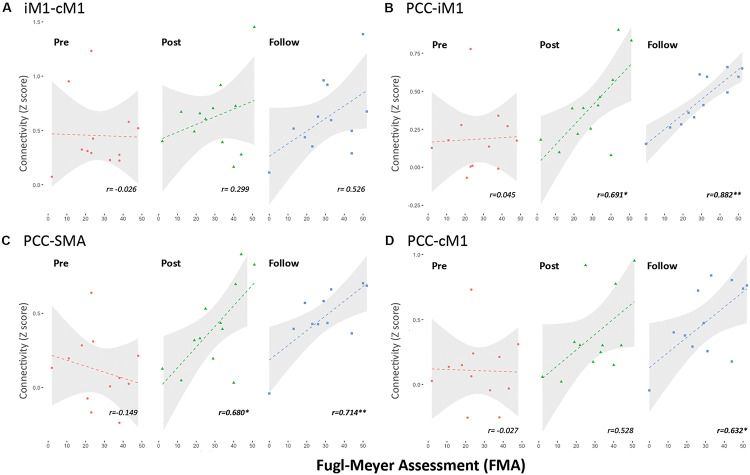
Association of FMA scores and functional connections in stroke rehabilitation. **(A)** Connection between iM1 and cM1 (within SMN). **(B)** Connection between PCC and iM1 (internetwork). **(C)** Connection between PCC and SMA (internetwork). **(D)** Connection between PCC and cM1 (internetwork). Connectivity strength was quantified by *z* score. Single asterisk sign denotes statistical significance *p* < 0.05, and double asterisk sign denotes *p* < 0.01.

Finally, we correlated the observed DMN–SMN connectivity with the motor-function improvements of FMA to establish their possible functional roles. Figure 6 illustrates the relationship between the FMA scores and the connectivity strengths (in *Z* score) of iM1 to cM1 (Figure 6A), PCC to iM1 (Figure 6B), PCC to SMA (Figure 6C), and PCC to cM1 (Figure 6D). Regarding the intra-network connectivity, the iM1-cM1 connection did not significantly correlate with FMA score at all times (*r*^2^ < 0.28, *p* > 0.05). Before the rehabilitation program started, the internetwork connectivity (PCC-iM1 and PCC-SMA) did not reveal its association with FMA scores before rehabilitation program (Pre: *r*^2^ < 0.06, *p* > 0.47), but it demonstrated significant associations right after the rehabilitation process (Post: *r*^2^ > 0.46, *p* < 0.015) and in the 1-month follow up (Follow: *r*^2^ > 0.51, *p* < 0.01). Besides, although the PCC-cM1 connection strength seemed enhanced after rehabilitation (Figure 5D), its association with FMA was only significant in the follow-up (Figure 6D).

## Discussion

We demonstrated the enhanced upper-limb motor performances (estimated by FMA and TEMPA) of stroke patients after the rehabilitation programs and 1-month follow-up. The working hypothesis was verified with significant internetwork interactions along the longitudinal rehabilitative assessments. Results indicated that the specific interactions between SMN and DMN emerged after stroke rehabilitation and could benefit the upper-limb motor functions. However, the FPN did not show significant internetwork interactions, nor did it facilitate motor performances in our observations. Furthermore, the brain activity in ipsilesional motor area recovered after rehabilitative training in correspondence with the emerging bilateral connectivity patterns in the Post and Follow states. These dynamic changes of brain functionality supported the concepts that the brain networks do not function independently but work as a whole especially in neurorehabilitation, and the inter-network synchronizations can reflect a compensatory effect of brain plasticity.

The stroke neurorehabilitation was evaluated using Task-fMRI, RS-fMRI, and clinical assessments in this study. In Task-fMRI, the task results of the affected-hand grasp showed no activation in SMN before rehabilitation (Figure 2A) and gradually returned back to ipsilesional activation in the patient group, resembling the brain activity in the NC. The recovered beta values in iM1 showed a positive relationship with the FMA scores (Figure 3A), indicating the effectiveness of the rehabilitation procedure. Relatively, the task results of the unaffected-hand grasp showed insignificant changes across the three time points (Figure 2B), suggesting the cM1 activity in the grasp task did not change along the rehabilitation process. In RS-fMRI, the most prominent changes across the longitudinal observations were on the SMN connectivity seeding at the affected side: the bilateral connectivity in NC was disrupted in stroke patients before rehabilitation and recovered back to bilateral connectivity after rehabilitation (Figure 2C). The recovered bilateral connectivity also showed significant association with the FMA scores (Figure 3B), demonstrating that both the asymmetricity in task activation and symmetricity in resting connectivity of SMN associated with the motor-function recovery in stroke patients. In contrast, the RSFC of DMN and FPN (Figures 2D,E) did not show significant differences across time, indicating that the stroke rehabilitation did not influence the within-network connectivity of DMN and FPN. Previous study showed that the impaired attention after stroke could recover by the cognitive rehabilitation (Lincoln et al., 2000). Nevertheless, the rehabilitation procedure in this study focused on motor function rather than cognitive training, which might lead to the unchanged RSFC within DMN and FPN.

The two functional indices, brain activity and connectivity, could both serve as neurophysiological indices reflecting the neurorehabilitation effects (Hu et al., 2017); however, they were seldom associated with each other in stroke patients. Wang et al. (2017) recently demonstrated the spatial similarity (coupling) between movement-induced activation and RSFC in stroke patients with motor impairment, and they concluded the similarity between motion-related activation and RSFC in the ipsilesional sensorimotor cortex was significantly increased following motor function recovery from the acute stage to the early chronic stage. Our results also demonstrated similar task/rest coupling phenomena in chronic stroke patients, indicating the neuroplasticity effect along the rehabilitation training beyond the effect of time after stroke onset. Furthermore, both task- and resting-state fMRI share similar principles of blood oxygenation level-dependent and neurovascular coupling (Tak et al., 2015), enabling the dual assessments for enhancing sensitivity of neuroplasticity in stroke rehabilitation. However, previous reports indicated the potential neurovascular uncoupling in stroke patients (Blicher et al., 2012), implying that the task-activation and RSFC in stroke patients shall not be inferred based on those indices in NCs. Beyond the recovery of network integration, an alternative inference was that the ipsilesional recovery of both grasp activity and SMN connectivity resulted from the transient effect of neurovascular uncoupling after stroke onset, and neurovascular coupling was sluggishly recovered following rehabilitation. At the current stage, we were unable to prove the neurorehabilitation effect was due to the recovery of network integrity or neurovasucalar coupling without collecting information on cerebrovascular reactivity.

The most prominent finding in this study was enhanced connections between PCC and iM1 after rehabilitation training. The interactions between DMN and SMN also showed a positive relationship with the motor-function performances, implying that DMN plays an important mediating role along stroke rehabilitation. A previous study showed decreased DMN RSFC associated with cognitive decline in stroke patients (Liu et al., 2014). Park et al. (2014) also demonstrated the decreased DMN connectivity at 1 month after stroke and restored at 3 months after stroke, suggesting a compensatory process for overcoming cognitive impairments. Similarly, our results also demonstrated a compensatory function of SMN-DMN interactions on motor performances along the stroke rehabilitation process. Nevertheless, these mentioned studies did not evaluate the rehabilitation process of included stroke patients, nor did they assess the cross-network interactions between DMN and SMN. Even though the current study could be the first report on the SMN–DMN interactions in stroke rehabilitation, the mechanism of SMN–DMN interactions remains elusive due to the unknown function behind the spontaneous synchronizations. Providing the presumption that the bilateral connectivity is vital to maintain cognitive functionality, we speculated that the transient breakdown of bilateral SMN connectivity following stroke onset would bypass through DMN regions to complete the inter-hemisphere synchronizations. This facilitatory effect of internetwork connectivity can emerge due to a time effect as suggested by Park et al. (2014) that the restoration of network connectivity was prominent between 1 and 3 months after stroke, but we regarded the beckoned internetwork facilitation due to the rehabilitation process. According to the previous observation of increased DMN connectivity after motor imagery learning (Ge et al., 2015), the specialized motor imagery training could enhance the motor functions through both physical action and motor imagination, reflecting on the SMN-DMN interactions correspondingly.

Due to the relatively strict inclusion and exclusion criteria for patient recruitment, we only completed the collection of 12 patients who voluntarily participated in the repeated measures over 3 months in past 4-year clinical practice. Beyond the limited sample size, the findings were confounded by the lesion site among stroke patients. In the current study, the lesion sites of the patients included in this study were confined to the subcortical regions to prevent major cortical damage without the chance of neuroplasticity. The lesion sites for each stroke patients were shown as highlighted masks overlaid on the anatomical image in Supplementary Figure S2. Third, we did not control the transient effects of medication because the six patients had been taking the medications prior to the first fMRI scan. Considering the medication effect, the hypertension medication could enhance the cerebral blood flow (CBF) globally without regional specificity (Matsumoto et al., 2009). The baclofen reduced the CBF of specific inferior brain regions (ventral striatum and medial orbitofrontal cortex), which were distant away from the findings in the current study (Franklin et al., 2011). Although the cloropidol administration was reported to increase the spatial extent of motor activation (Chang, 2012), the motor activation of the affected hand was opposite from the increased spatial extent, and the network interaction was less likely contributed from the single patient who took cloropidol. Therefore, we regarded that the medication had minimal impacts on our findings. Another limitation in this study was the focus on the evaluation of motor functions in stroke rehabilitation, and henceforth, the other cognitive assessments were not included. A previous study demonstrated the attention deficits following stroke (Lincoln et al., 2000; Bajaj et al., 2015), but we did not observe the alterations of FPN connectivity along the stroke rehabilitation. Without the cognitive evaluations, we were unable to provide a corresponding inference based on the existing materials. Targeting on chronic stroke patients with subcortical lesions, we performed longitudinal investigations on patients’ motor functions (evaluated by FMA) and the underlying neural mechanisms (using Task-/RS-fMRI techniques) along the rehabilitation process. Results disclosed that the DMN, especially in PCC, did not show significant synchronizations with SMN before the rehabilitation process, nor did it appear in the NC group. After the 24-h rehabilitation process, the dynamically enhanced internetwork interactions associated with patients’ motor performances (assessed by FMA) in stroke rehabilitation, implying a facilitative effect of PCC synchronization on the upper-limb motor functions.

## Conclusion

The longitudinal measures of brain activity and connectivity revealed the neuroplasticity in stroke rehabilitation. Not only did the motor activity and connectivity gradually recover back to a relatively normal condition within the SMN, but between-network interactions were also involved in the rehabilitation process. Before rehabilitation, the cross-network interaction was low before the rehabilitation process, similar to the level of NCs. After rehabilitation, the enhanced PCC-SMN connectivity facilitates the motor recovery after stroke rehabilitation. Our findings suggested that PCC-SMN connectivity plays a facilitatory role following the stroke rehabilitation, which can be served as a neurorehabilitative biomarker for future intervention evaluations.

## Data Availability Statement

The datasets generated for this study are available on request to the corresponding author.

## Ethics Statement

All procedures performed in studies involving human participants were in accordance with the ethical standards of the institutional and/or national research committee and with the 1964 Helsinki declaration and its later amendments or comparable ethical standards. All protocols were under the supervision of Institutional Review Board of Taipei Veterans General Hospital (IRB number: 2013-05-018A). Informed consents were obtained from all participants included in the study.

## Author Contributions

C-CC, S-HL, and CW contributed to conception of the work. S-HL, S-CY, C-CC, and S-HL contributed to experimental design and data acquisition. S-HL, L-MH, CW, and S-FG contributed to data analysis. S-HL and CW contributed to interpretation of the finding.

## Conflict of Interest

The authors declare that the research was conducted in the absence of any commercial or financial relationships that could be construed as a potential conflict of interest.

## References

[B1] Bajaj S. Butler A. J. Drake D. Dhamala M. (2015). Functional organization and restoration of the brain motor-execution network after stroke and rehabilitation. *Front. Hum. Neurosci.* 9:173. 10.3389/fnhum.2015.00173 25870557 PMC4378298

[B2] Biswal B. Zerrin Yetkin F. Haughton V. M. Hyde J. S. (1995). Functional connectivity in the motor cortex of resting human brain using echo-planar mri. *Magn. Reson. Med.* 34 537–541. 10.1002/mrm.1910340409 8524021

[B3] Blicher J. U. Stagg C. J. O’Shea J. Østergaard L. MacIntosh B. J. Johansen-Berg H. (2012). Visualization of altered neurovascular coupling in chronic stroke patients using multimodal functional MRI. *J Cereb Blood Flow Metab* 32 2044–2054. 10.1038/jcbfm.2012.105 22828998 PMC3493993

[B4] Bushnell C. Bettger J. P. Cockroft K. M. Cramer S. C. Edelen M. O. Hanley D. (2015). Chronic stroke outcome measures for motor function intervention trials: expert panel recommendations. *Circ. Cardiovasc. Qual. Outcomes* 8 S163–S169. 10.1161/CIRCOUTCOMES.115.002098 26515205 PMC5289112

[B5] Carey L. M. (2012). *Stroke Rehabilitation.* Oxford: Oxford University Press.

[B6] Carey L. M. Abbott D. F. Harvey M. R. Puce A. Seitz R. J. Donnan G. A. (2011). Relationship between touch impairment and brain activation after lesions of subcortical and cortical somatosensory regions. *Neurorehabil. Neural. Repair* 25 443–457. 10.1177/1545968310395777 21382887

[B7] Carey L. Macdonell R. Matyas T. A. (2011). SENSe: study of the effectiveness of neurorehabilitation on sensation: a randomized controlled trial. *Neurorehabil. Neural. Repair.* 25 304–313. 10.1177/1545968310397705 21350049

[B8] Carey L. M. Seitz R. J. (2007). Functional neuroimaging in stroke recovery and neurorehabilitation: conceptual issues and perspectives. *Int. J. Stroke* 2 245–264. 10.1111/j.1747-4949.2007.00164.x 18705925

[B9] Carter A. R. Astafiev S. V. Lang C. E. Connor L. T. Rengachary J. Strube M. J. (2010). Resting interhemispheric functional magnetic resonance imaging connectivity predicts performance after stroke. *Ann. Neurol.* 67 365–375. 10.1002/ana.21905 20373348 PMC2927671

[B10] Chang Y. (2012). Pharmacological functional magnetic resonance imaging of cloropidol on motor Task. *J. Korean Soc. Mag. Resonance Med.* 16 136–141. 10.13104/jksmrm.2012.16.2.136

[B11] Cox R. W. (1996). AFNI: software for analysis and visualization of functional magnetic resonance neuroimages. *Comput. Biomed. Res.* 29 162–173. 10.1006/cbmr.1996.0014 8812068

[B12] Crum R. M. Anthony J. C. Bassett S. S. Folstein M. F. (1993). Population-based norms for the mini-mental state examination by age and educational level. *JAMA* 269 2386–2391. 8479064

[B13] Desrosiers J. Hebert R. Bravo G. Dutil E. (1995). Upper extremity performance test for the elderly (TEMPA): normative data and correlates with sensorimotor parameters. test d’Evaluation des membres supérieurs de personnes agées. *Arch. Phys. Med. Rehabil.* 76 1125–1129. 8540788 10.1016/s0003-9993(95)80120-0

[B14] Enzinger C. Johansen-Berg H. Dawes H. Bogdanovic M. Collett J. Guy C. (2008). Functional MRI correlates of lower limb function in stroke victims with gait impairment. *Stroke* 39 1507–1513. 10.1161/STROKEAHA.107.501999 18340092 PMC7610857

[B15] Fan Y.-T. Wu C.-Y. Liu H.-L. Lin K.-C. Wai Y.-Y. Chen Y.-L. (2015). Neuroplastic changes in resting-state functional connectivity after stroke rehabilitation. *Front. Hum. Neurosci.* 9:546. 10.3389/fnhum.2015.00546 26557065 PMC4617387

[B16] Franklin T. R. Wang Z. Sciortino N. Harper D. Li Y. Hakun J. (2011). Modulation of resting brain cerebral blood flow by the GABA B agonist, baclofen: a longitudinal perfusion fMRI study. *Drug Alcohol. Depend* 117 176–183. 10.1016/j.drugalcdep.2011.01.015 21333466 PMC3348615

[B17] Fugl-Meyer A. R. Jääskö L. Leyman I. Olsson S. Steglind S. (1975). The post-stroke hemiplegic patient. 1. a method for evaluation of physical performance. *Scand. J. Rehabil. Med.* 7 13–31. 1135616

[B18] Ge R. Zhang H. Yao L. Long Z. (2015). Motor imagery learning induced changes in functional connectivity of the default mode network. *IEEE Trans. Neural. Syst. Rehabil. Eng.* 23 138–148. 10.1109/TNSRE.2014.2332353 25014958

[B19] Hsieh F.-I. Chiou H.-Y. (2014). Stroke: morbidity, risk factors, and care in taiwan. *J. Stroke* 16 59–64. 10.5853/jos.2014.16.2.59 24949310 PMC4060269

[B20] Hu J. Du J. Xu Q. Yang F. Zeng F. Dai X.-J. (2017). Altered coupling between motion-related activation and resting-state brain activity in the ipsilesional sensorimotor cortex after cerebral stroke. *Front. Neurol.* 8:339. 10.3389/fneur.2017.00339 28769870 PMC5515815

[B21] Kolb B. Teskey G. C. Gibb R. (2010). Factors influencing cerebral plasticity in the normal and injured brain. *Front. Hum. Neurosci.* 4:204. 10.3389/fnhum.2010.00204 21120136 PMC2991189

[B22] Lam T. K. Dawson D. R. Honjo K. Ross B. Binns M. A. Stuss D. T. (2018). Neural coupling between contralesional motor and frontoparietal networks correlates with motor ability in individuals with chronic stroke. *J. Neurol. Sci.* 384 21–29. 10.1016/j.jns.2017.11.007 29249372

[B23] Li S. Ma Z. Tu S. Zhou M. Chen S. Guo Z. (2014). Altered resting-state functional and white matter tract connectivity in stroke patients with dysphagia. *Neurorehabil. Neural. Repair.* 28 260–272. 10.1177/1545968313508227 24297761

[B24] Lin C.-J. Tu P.-C. Chern C.-M. Hsiao F.-J. Chang F.-C. Cheng H.-L. (2014). Connectivity features for identifying cognitive impairment in presymptomatic carotid stenosis. *PLoS One* 9:e85441. 10.1371/journal.pone.0085441 24454868 PMC3893296

[B25] Lincoln N. B. Majid M. J. Weyman N. (2000). Cognitive rehabilitation for attention deficits following stroke. *Cochrane Database Syst. Rev.* 7:CD002842. 10.1002/14651858.CD002842 11034773

[B26] Liu J. Qin W. Wang H. Zhang J. Xue R. Zhang X. (2014). Altered spontaneous activity in the default-mode network and cognitive decline in chronic subcortical stroke. *J. Neurol. Sci.* 347 193–198. 10.1016/j.jns.2014.08.049 25304057

[B27] Liu J. Wang Q. Liu F. Song H. Liang X. Lin Z. (2017). Altered functional connectivity in patients with post-stroke memory impairment: a resting fMRI study. *Exp. Ther. Med.* 14 1919–1928. 10.3892/etm.2017.4751 28962104 PMC5609161

[B28] Matsumoto S. Shimodozono M. Miyata R. Kawahira K. (2009). Effect of the angiotensin II type 1 receptor antagonist olmesartan on cerebral hemodynamics and rehabilitation outcomes in hypertensive post-stroke patients. *Brain Inj.* 23 1065–1072. 10.3109/02699050903379404 19891533

[B29] Nowak D. A. Grefkes C. Ameli M. Fink G. R. (2009). Interhemispheric competition after stroke: brain stimulation to enhance recovery of function of the affected hand. *Neurorehabil. Neural. Repair.* 23 641–656. 10.1177/1545968309336661 19531606

[B30] Park J.-Y. Kim Y.-H. Chang W. H. Park C.-H. Shin Y.-I. Kim S. T. (2014). Significance of longitudinal changes in the default-mode network for cognitive recovery after stroke. *Eur. J. Neurosci.* 40 2715–2722. 10.1111/ejn.12640 24931140

[B31] Ripollés P. Marco-Pallarés J. de Diego-Balaguer R. Miró J. Falip M. Juncadella M. (2012). Analysis of automated methods for spatial normalization of lesioned brains. *NeuroImage* 60 1296–1306. 10.1016/j.neuroimage.2012.01.094 22305954

[B32] Seghier M. L. Ramlackhansingh A. Crinion J. Leff A. P. Price C. J. (2008). Lesion identification using unified segmentation-normalisation models and fuzzy clustering. *NeuroImage* 41 1253–1266. 10.1016/j.neuroimage.2008.03.028 18482850 PMC2724121

[B33] Tak S. Polimeni J. R. Wang D. J. J. Yan L. Chen J. J. (2015). Associations of resting-state fMRI functional connectivity with flow-BOLD coupling and regional vasculature. *Brain Connect* 5 137–146. 10.1089/brain.2014.0299 25384681 PMC4394176

[B34] Tuladhar A. M. Snaphaan L. Shumskaya E. Rijpkema M. Fernandez G. Norris D. G. (2013). Default mode network connectivity in stroke patients. *PLoS One* 8:e66556. 10.1371/journal.pone.0066556 23824302 PMC3688936

[B35] Van Dijk K. R. A. Hedden T. Venkataraman A. Evans K. C. Lazar S. W. Buckner R. L. (2010). Intrinsic functional connectivity as a tool for human connectomics: theory. Properties, and optimization. *J. Neurophysiol.* 103 297–321. 10.1152/jn.00783.2009 PMC2807224 19889849

[B36] van Meer M. P. A. Otte W. M. van der Marel K. Nijboer C. H. Kavelaars A. van der Sprenkel J. W. B. (2012). Extent of bilateral neuronal network reorganization and functional recovery in relation to stroke severity. *J. Neurosci.* 32 4495–4507. 10.1523/JNEUROSCI.3662-11.2012 22457497 PMC6622065

[B37] van Meer M. P. A. van der Marel K. Wang K. Otte W. M. Ei Bouazati S. Roeling T. A. P. (2010). Recovery of sensorimotor function after experimental stroke correlates with restoration of resting-state interhemispheric functional connectivity. *J. Neurosci.* 30 3964–3972. 10.1523/JNEUROSCI.5709-09.2010 20237267 PMC6632290

[B38] Veldsman M. Curwood E. Pathak S. Werden E. Brodtmann A. (2018). Default mode network neurodegeneration reveals the remote effects of ischaemic stroke. *J. Neurol. Neurosurg. Psychiatry* 89 318–320. 10.1136/jnnp-2017-315676 28747402

[B39] Wang C. Qin W. Zhang J. Tian T. Li Y. Meng L. (2014). Altered functional organization within and between resting-state networks in chronic subcortical infarction. *J. Cereb. Blood Flow Metab.* 34 597–605. 10.1038/jcbfm.2013.238 24398939 PMC3982082

[B40] Wang T. Xiao F. Wu G. Fang J. Sun Z. Feng H. (2017). Impairments in brain perfusion, metabolites, functional connectivity, and cognition in severe asymptomatic carotid stenosis patients: an integrated MRI study. *Neural. Plasticity* 2017 1–7. 10.1155/2017/8738714 28255464 PMC5309400

[B41] Wolf S. L. Catlin P. A. Ellis M. Archer A. L. Morgan B. Piacentino A. (2001). Assessing wolf motor function test as outcome measure for research in patients after stroke. *Stroke* 32 1635–1639. 10.1161/01.STR.32.7.1635 11441212

[B42] Zhang Y. Liu H. Wang L. Yang J. Yan R. Zhang J. (2016). Relationship between functional connectivity and motor function assessment in stroke patients with hemiplegia: a resting-state functional MRI study. *Neuroradiology* 58 503–511. 10.1007/s00234-016-1646-5 26843179

