# Supplementary material for: Synchrony Between Default-Mode and Sensorimotor Networks Facilitates Motor Function in Stroke Rehabilitation: A Pilot fMRI Study
Source: Front Neurosci. 2020 Jun 16;14:548. doi: 10.3389/fnins.2020.00548 (PMC7325875; doi:10.3389/fnins.2020.00548)
Supplement: Supplementary file 1 [file Table_1.DOCX]

**Supplementary Information**


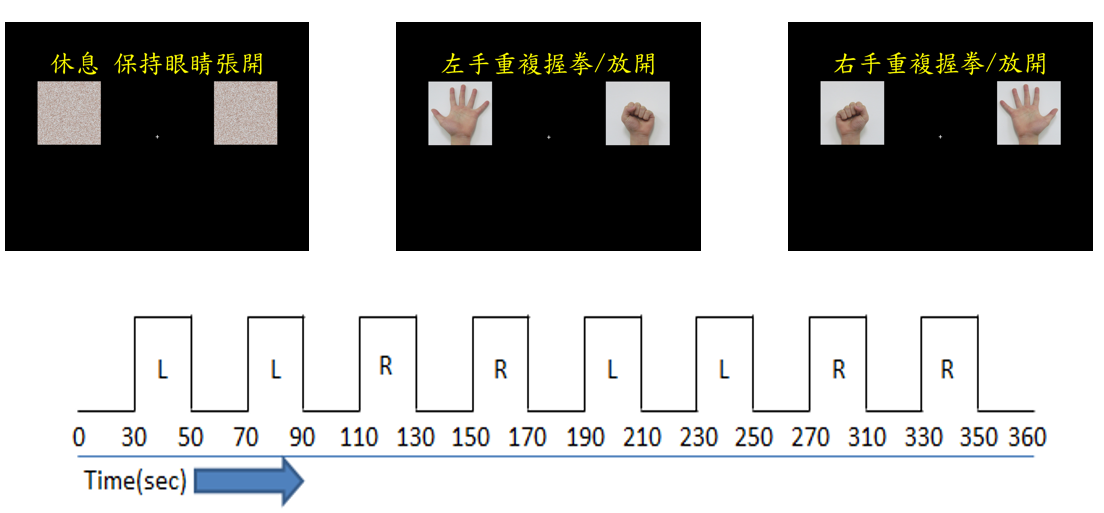


**Figure S1** The visual instruction of grasp task for the task-fMRI scanning session. The patients were instructed to maintain rest without hand movements (left panel), grasping by left hand (middle panel) and the grasping using right hand (right panel). During grasping, the subject was instructed to grasp at their maximum force in executing the hand movement. Grasping force was not measured during the task. During resting, the presented two photos on screen were the scrambled photos used in the hand-movement used in grasping to fixate the light exposure of visual stimuli.


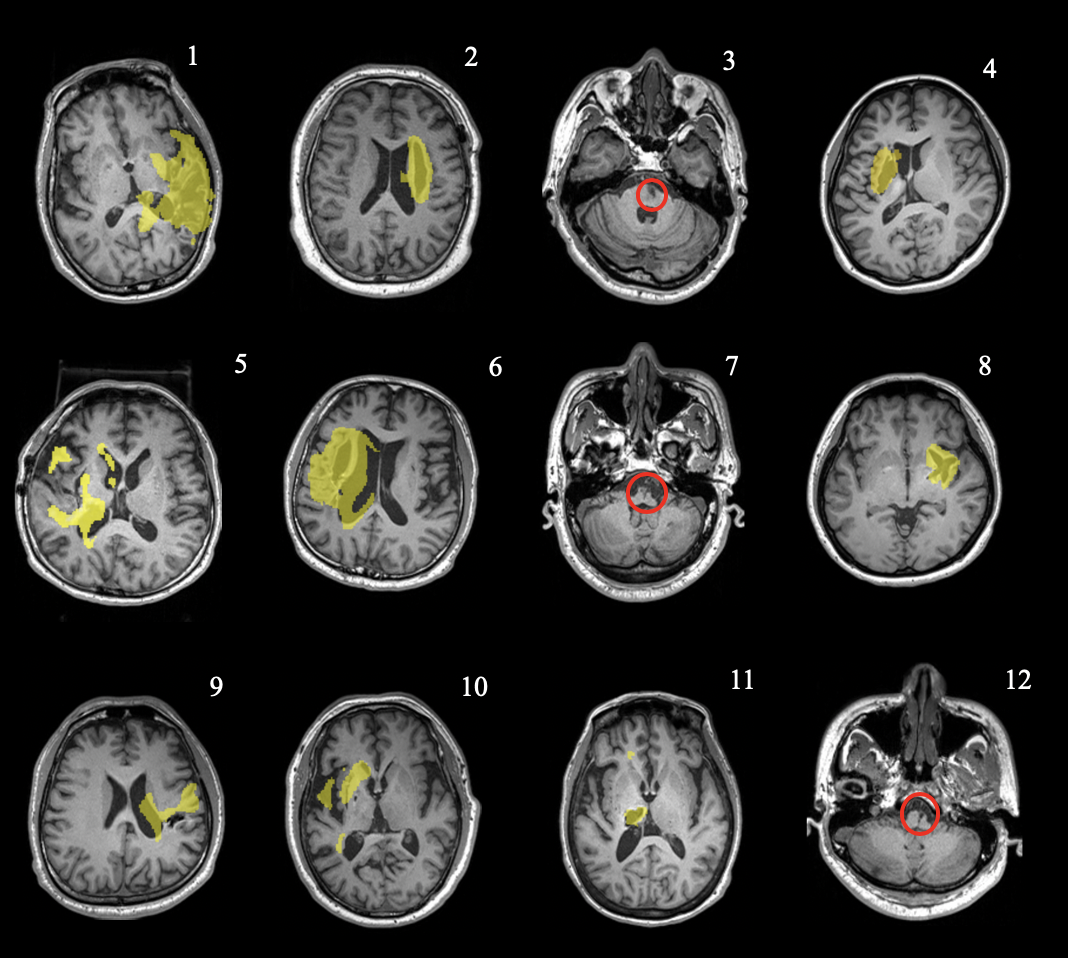


**Figure S2.** T1-weighted images overlaid by the lesion mask for each patient. The lesion mask was highlighted by yellow masks generated using ALI toolbox for all stroke patients. For patient number 3, 7 and 12, red circles are presented in replacement of lesion masks due to the lesion location outside of the field of view in fMRI datasets.

# TABLE

**Table S1** Demographic and clinical characteristics of both normal control and stroke patients (Mean±SEM).

|  | Normal control (n = 12) | Stroke Patient  (n = 12) | *T / χ^2^* | *p*-value |
| --- | --- | --- | --- | --- |
| Age | 43.5±3.4 | 46.2±3.4 | T = 0.55 | 0.59^a^ |
| Education | 15.7±1.3 | 12.8±0.8 | T = 1.79 | 0.09^a^ |
| Male (%) | 5 (42%) | 10 (83%) | χ^2^=2.84 | 0.09^b^ |
| Right-handedness (%) | 12 (100%) | 12 (100%) | 0 | 1 |
| Time after stroke onset (months) | -- | 12.9±1.7 |  |  |

^a^ Two-sample *t*-test

^b^ Chi-squared test with continuity correction

# Table S2. Demographic characteristics of stroke patients included in this study.

| ID | Lesion site | Affected side | Gender | Age | Educa-tion (years) | Month after stroke | Brunnstrom stage (proximal/ distal) | FMA  (pre:post: follow) | WMFT  (pre:post: follow) | TEMPA  (pre:post: follow) |
| --- | --- | --- | --- | --- | --- | --- | --- | --- | --- | --- |
| 1 | MCA | Right | Male | 57 | 12 | 8 | 3/3 | 24:33:31 | 38:54:49 | -105:-87:-67 |
| 2 | Basal ganglia | Right | Male | 34 | 14 | 10 | 5/4 | 38:40:44 | 49:60:48 | -81:-82:-81 |
| 3 | Paramedian pontine | Right | Male | 44 | 14 | 24 | 4/5 | 11:12:13 | 37:36:39 | -15:-5:-4 |
| 4 | Basal ganglia | Left | Female | 27 | 12 | 13 | 4/5 | 33:34:33 | 46:45:47 | -68:-65:-50 |
| 5 | Basal ganglia | Left | Male | 55 | 18 | 12 | 3/3 | 18:19:19 | 39:39:32 | -77:-67:-64 |
| 6 | Basal ganglia | Left | Male | 44 | 12 | 25 | 2/2 | 2:2:0 | 18:10:11 | -40:-33:-41 |
| 7 | Medulla | Right | Male | 46 | 14 | 12 | 5/5 | 43:44:44 | 62:65:63 | -23:-28:-23 |
| 8 | Basal ganglia | Left | Male | 54 | 12 | 6 | 5/5 | 48:51:50 | 72:69:71 | -60:-52:-45 |
| 9 | MCA |  | Male | 31 | 14 | 14 | 3/4 | 23:29:29 | 40:40:39 | -82:-79:-73 |
| 10 | MCA | Left | Male | 63 | 12 | 15 | 4/3 | 21:22:23 | 37:37:38 | -63:-63:-63 |
| 11 | Basal ganglia | Left | Female | 60 | 6 | 8 | 2/4 | 23:25:26 | 36:48:42 | -88:-88:-84 |
| 12 | Medulla | Right | Male | 39 | 14 | 8 | 5/4 | 38:41:52 | 72:73:74 | -16:-14:-7 |

MCA: middle cerebral artery territory
